# The Origin of RNA and the Formose–Ribose–RNA Pathway

**DOI:** 10.3390/ijms25126727

**Published:** 2024-06-19

**Authors:** Gaspar Banfalvi

**Affiliations:** Department of Molecular Biotechnology and Microbiology, University of Debrecen, 4032 Debrecen, Hungary; banfalvi.gaspar@science.unideb.hu or gaspar.banfalvi@gmail.com; Tel.: +36-52-512-900 (ext. 623190) or +36-20-580-8325; Fax: +36-52-512-925

**Keywords:** modell building, nucleotides structure, why ribose was selected, formose reactions, ribose phosphorylation, ribose phosphate polymerization, pre RNA formation, from preRNA to genRNA, first metabolic pathway, DNA Empire

## Abstract

Prebiotic pre-Darwinian reactions continued throughout biochemical or Darwinian evolution. Early chemical processes could have occurred on Earth between 4.5 and 3.6 billion years ago when cellular life was about to come into being. Pre-Darwinian evolution assumes the development of hereditary elements but does not regard them as self-organizing processes. The presence of biochemical self-organization after the pre-Darwinian evolution did not justify distinguishing between different types of evolution. From the many possible solutions, evolution selected from among those stable reactions that led to catalytic networks, and under gradually changing external conditions produced a reproducible, yet constantly evolving and adaptable, living system. Major abiotic factors included sunlight, precipitation, air, minerals, soil and the Earth’s atmosphere, hydrosphere and lithosphere. Abiotic sources of chemicals contributed to the formation of prebiotic RNA, the development of genetic RNA, the RNA World and the initial life forms on Earth and the transition of genRNA to the DNA Empire, and eventually to the multitude of life forms today. The transition from the RNA World to the DNA Empire generated new processes such as oxygenic photosynthesis and the hierarchical arrangement of processes involved in the transfer of genetic information. The objective of this work is to unite earlier work dealing with the formose, the origin and synthesis of ribose and RNA reactions that were published as a series of independent reactions. These reactions are now regarded as the first metabolic pathway.

## 1. Introduction

Life is a self-sustained chemical system capable of undergoing Darwinian evolution [1]. Self-sustaining refers to utilizing the energy of the environment for programmed anabolic and catabolic reactions. The chain of continuously extended consecutive reactions is termed the metabolic pathway. The initial interactions of metabolic and genetic reactions are centered around the CHNOPS group of elements in the periodic table with the irreplaceable role of hydrogen, oxygen nitrogen and carbon and their simple compounds consisting of the three-atom-containing HCNs that are in space, water on Earth, the most abundant compound, and the five atoms contained in the hydrocarbon methane (CH4). The basic reactions between the compounds in the CHNOPS group are regarded as the first abiotic reactions. The reaction of hydrogen cyanide with water results in formamide (H_2_NCOH). The availability of HCN was significantly limited on Hadean Earth.

The origin of life has been continuously studied in an effort to understand how chemical reactions could have resulted in a primordial cell system [2]. Several theories have tried to answer the question of how disordered chemical reactions developed into a highly organized metabolic pathway. Pre-Darwinian evolution is referred to as chemical evolution, whereas Darwinian evolution corresponds to the encoded genetic information upon which natural selection acts [3]. This definition does not take into consideration that Darwinian evolution is not equal to biological evolution, as the preference is often overridden by non-selective factors such as growing populations of a small size and varying trait compositions. The distinction among different kinds of evolution would mean that processes could occur in more than one direction even though there is no evidence for pre- or post-cellular life nor any indication that other non-cellular life forms would have existed or replaced the ribonucleic acid-based life. It is more tangible to assume that evolution progresses step-by-step by incorporating useful genetic information. This does not mean that several promising solutions would not have failed even if their temporarily promising perspectives proved to be dead ends. There is no agreement among scientists regarding the appearance of preRNA and coding genetic RNA (genRNA) or how this transition could have taken place. Even the supporters of the RNA World hypothesis questioned whether the RNA World took a relatively short time to form (up to 100 million years) or a longer period that could have lasted up to billions of years.

Despite its popularity, there are arguments against the concept of the RNA World and the gap between the appearance of genRNA and the bacterial World. Geochemical evidence supports that prebiotic RNA life could have existed before 3.7–3.8 billion years ago [4,5]. The pessimistic view is that oxygenic photosynthesizing cyanobacteria may have originated 3 billion years ago [6]. The more optimistic fossil evidence of cyanobacterial oxygen production extends back to 3.6–3.7 billion years ago [7]. Radioisotope dating points out that extremophile cellular metabolism existed even earlier, reflecting a carbon-based self-replicating microbial life. The comparison of the age of the Earth (4.54 years ago [8,9,10] with the appearance of bacterial life under extreme conditions suggested that a narrow window of time was left for the RNA World to come into being. There is an agreement that bacteria and archeobacteria under extreme conditions could have developed from a common ancestor four billion years ago in a reducing atmosphere. The fossils of oxygen-producing blue-green bacteria (cyanobacteria) are about four billion years old and they were among the earliest group of bacteria. The sudden rise in the atmospheric oxygen levels about 2.32–2.45 billion years ago [11] is known as the Great Oxygenation Event [12]. References related to cyanobacteria suggest that the RNA World lasted longer than expected. The oldest abiotic reactions could have occurred in an aqueous solution, sorbed to surfaces or synthetic processes could have taken place in dry intermountain valleys. Reaction rates depended on the reaction mechanisms, chemical structures, and relative concentrations of catalysts such as protons, hydroxyl ions, transition metals, and clay particles. This review describes a new concept of the origin of preRNA and how genRNA was formed by testing the plausibility and taking into consideration the opinions of several other scientists.

### 1.1. Abiotic Reactions

**Figure 1:**
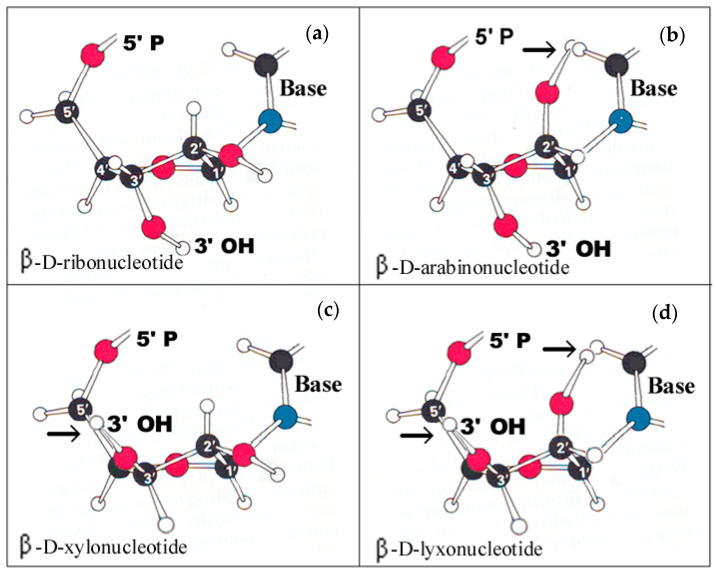
Demonstration of β-D-ribose being the best and lyxose the least fitting pentose in nucleotide building blocks of nucleic acids. (**a**) In ribonucleotides, the bulky base at C1′ and the phosphate group at C5′ position are distantly located from each other to be outside the van der Waals distance. In ribose, the C2′ and C3′ hydroxyl groups are on the opposite side of the pentose ring. Due to the major C2′-endo and the minor C3′-exo conformation the hydroxyl groups of the C2′ and C3′ point in different directions and are far enough apart to allow free rotation. (**b**) In arabinonucleotide, the C2′ hydroxyl is above the plane of the ring but the base falls slightly within van der Waals distance relative to the C2′-OH. The spherical hindrance of these groups prevents the free rotation, as is shown by the upper right black arrow in this panel. (**c**) Xylonucleotide: the steric vicinity of C3′ and C5′ substituents hinders the free rotation shown by the black arrow at the left side of this panel. (**d**) Lyxonucleotide: all functional groups would be squeezed inside the cage above the plane of the ring. The spatial incompatibility is caused by the lack of space. Free rotation is prevented by the C5′-phosphate, C3′-OH and C2′-OH, as shown by the black arrows [20]. Reproduced with permission from the free PMC article from Banfalvi, G. “Why ribose was selected as the sugar component of nucleic acids”. *DNA Cell Biol.*, 2006, Figure 5.

Abiotic reactions were imitated experimentally by trying to produce substantial quantities of precursors to claim that a prebiotic synthesis of these reactions could have occurred on the Earth some four billion years ago. The research into abiotic reactions focused on those reactions that were identified as including key compounds of our biosystem including amino acids, sugary substances, sugars, formose sugars, nucleobases, purine and pyrimidine ribonucleotides, etc. [11,12,13,14,15,16,17,18,19,20,21,22]. For abiotic RNA formation, there are simple compounds, the reactions of which could be used for its synthesis. Nucleic acids contain only a single pentose (ß-D ribose in RNA and ß-D-deoxyribose in DNA) that fits perfectly into the nucleotide units and can freely rotate around its axis and secure maximal flexibility for nucleic acids [20]. The α-anomeric ribose forms an α-D-ribopyranose ring consisting of six atoms. (The cyclization of ß D-ribose occurs via hemiacetal formation, containing six atoms forming a ring structure of five carbon atoms and one oxygen atom (β-D-ribose). β-D-ribose formed in the formose reaction by the polymerization of formaldehyde is still the only known reaction of prebiotic ribose synthesis [23], indicating that it could not be replaced by other sugars. The formose reaction has been proposed as an old metabolic pathway. Coupling it with prebiotic RNA and then to genetic RNA synthesis, contributed to the establishment of the RNA World hypothesis, to life on Earth and more importantly led to the transition from the RNA Word to the DNA Empire. The connection of small biogenic compounds into long chains resulted in informational macromolecules. To store genetic information in macromolecular RNA was a challenging task since the nucleotide building blocks are by themselves complex compounds consisting of three parts (sugar, phosphate, nucleobase). One of the frequently raised questions was why is the sugar component of the nucleotide building blocks a pentose, specifically ß-D ribose? For the selection of ribose as a sugar component of nucleic acids, molecular modelling provided the clue, revealing that of the four pentoses, only the β anomeric D-ribose could be inserted into the nucleotides without losing the rotational freedom of the sugar and other functional groups (OH, phosphate, nucleobase) and securing the flexibility of the nucleic acid structure Figure 1 [20].

Figure 1 demonstrates that ribose selection was not a random process but the only possible solution as only β-D-ribose fits perfectly into the ribonucleotide structure. Trying to fit any other pentose into the nucleotide will encounter van der Waals forces preventing the free rotation of substituents of the pentoses around the sigma bonds. The steric hindrance imposed is indicated by black arrows in Figure 1.

### 1.2. Arabinonucleotides in Double-Stranded DNA

**Figure 2:**
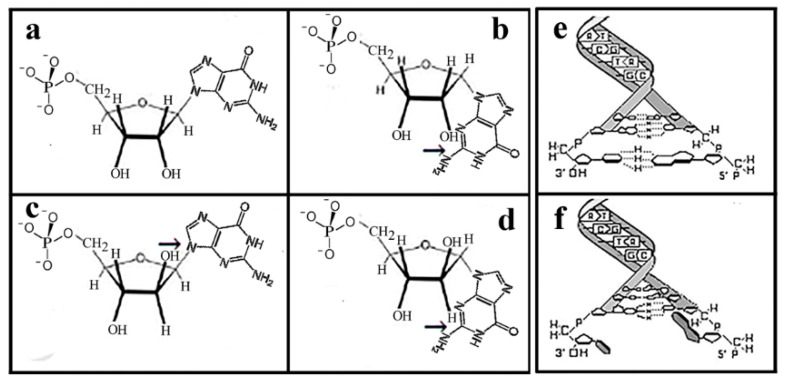
Demonstration of anomeric glycosidic bonds of nucleotides impacting the formation of the helical structure. (**a**) In β-D ribonucleotides, the anomeric bonds are in β position, far enough to secure free rotation. (**b**) In the α-D-ribonucleotide, the anomeric bond is within Van der Waals distance between the base and sugar preventing free rotation. (**c**) β-D arabinonucleotide: the 2′deoxy H-bond and base are too close and prevent free rotation indicated with the black arrow. (**d**) Lack of double-stranded structure formation in DNA containing exclusively α-D-ribose. (**e**) Base pairing and double DNA structure formed in the presence of ribonucleotides. (**f**) Lack of base pairing at the lower end of the DNA structure in the presence of ‘one pair of α-D-arabinose. Reproduced with permission from Banfalvi, G. “Prebiotic Pathway from ribose to RNA formation”. *Int. J. Mol. Sci.*, 2021, Figure 2.

A further restriction regarding the free rotation of nucleotides in DNA is related to the presence of the α-anomeric glycosidic bond (Figure 2). A schematic presenting why only β-D ribonucleotides but not other pentose-containing nucleotides can form double-stranded helical structures is shown in Figure 2. In DNA containing only β-D-ribose (Figure 2a) the anomeric sugar-base glycosidic bonds the anomeric glycosylic connection is β. This means that the nucleobases are as distant as possible from the ribose to secure the free rotation of the sugars and bring the bases at the opposite sides of the two chains close to each other for hydrogen base pairing in the middle of the helical structure.

## 2. The Formose–Ribose–RNA Pathway

The example of connecting three parts of related reactions into one pathway does not mean that this would be the only prebiotic route. Although the connection from ribose to other reactions is not indicated, it can go to nucleotides, other pentoses, hexoses, hexose phosphates, glycolysis, and the pentose phosphate cycle, just to mention the best-known ones.

**Figure 3:**
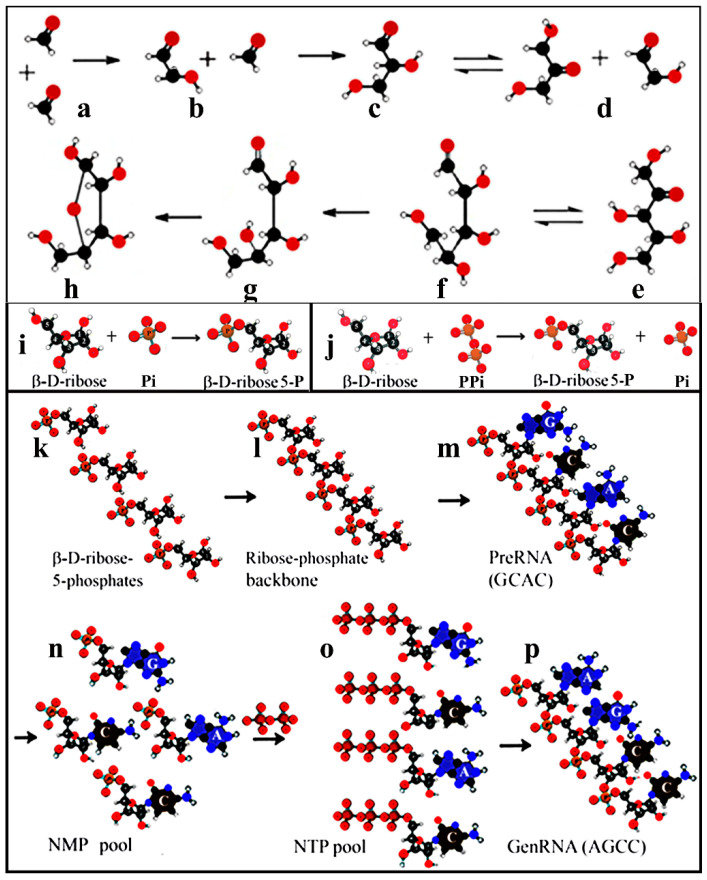
The formose–ribose–RNA pathway. The upper box contains the formose reaction starting with (**a**) two formaldehyde molecules, and (**b**) condensed to glycolaldehyde. (**c**) The subsequent aldol reaction extended the chain length by two carbon units, forming glyceraldehyde (**d**) that underwent an aldose–ketose isomerization. (**e**) Glyceraldehyde reacts with glycolaldehyde to bring about pentulose. (**f**) Selective isomerization takes place between pentulose (**e**) and ribose (**f**) favoring the nucleophilic addition of D-ribose (**g**) and its ring formation to D-ribose (**h**). The nucleophylic reaction produces racemic ribose. Enantioselective β-D-ribose is depicted only to show its further metabolism in the formose–ribose–RNA pathway. Reproduced with permission from Banfalvi, G. “Prebiotic Pathway from ribose to RNA formation”. *Int. J. Mol. Sci.*, 2021, Figures 1 and 3.

In Figure 3i,j, β-D-ribose is phosphorylated to β-D-ribose 5-phosphate. The lower box in Figure 3 contains Figure 3k which shows ribose phosphates polymerized to the ribose–phosphate backbone (Figure 3l), to which nucleobases can be attached in a random manner giving rise to preRNA (Figure 3m). The rest of the lower box of Figure 3 contains hydrolyzed preRNA (Figure 3n) phosphorylated to NTPs (Figure 3o), serving as substrates for genRNA synthesis (Figure 3p).

The formation of prebiotic RNA assumes that ribonucleoside triphosphate substrates were not available on prebiotic Earth and became the building blocks of RNA after genRNA was established. One of the most debated questions concerns the availability and synthesis of prebiotic ribose. One of the oldest theories about ribose formation comes from the formose reaction [21]. The low yield of ribose [22,23] works against the mass action law and does not explain how the macromolecular synthesis of RNA could have been initiated. In addition, the ester linkage between ribose and phosphoric acid in RNA is prone to hydrolysis [24]. The low yield of ribose [25,26,27] could have been elevated by the protective and stabilizing effect of borate-containing minerals [28,29], by silicate, phosphate and calcium [30,31,32], and by adduct formation with cyanamide. These observations questioned the one-pot synthesis of nucleotides and strengthened the assumption that significant quantities of compounds could have come from space such as photo-processed phosphate from cosmic ice [33].

Orgel studied the accumulation of the ribose phosphate units but was skeptical about self-sustaining metabolic pathways that could spontaneously come into existence on the early Earth and evolve into life, since metabolic pathways have little reason to evolve into genetic molecules without becoming even more complex. The missing link in Orgel’s pathway [30] was the phosphorylation of ribose [31] (Figure 3i,j). The next step could have been the alignment of ribose phosphates (Figure 3k) that by polymerization brought about the sugar–phosphate backbone (Figure 3l). It is logical to assume that self-sustaining metabolic pathways could not develop spontaneously, and rather a step–by–step synthesis could have taken place which was often coupled to side reactions rather than connected to metabolic cycles to continue the metabolic pathway consisting of linear, cyclic and spiral metabolic routes. The combination of cycles into a so-called metabolic clockwork generates new cycles. In the metabolic clockwork, the central element is the citrate cycle and most of the secondary cycles are coupled to this central wheel [34]. Cycles generate cycles and the eighteen known metabolic cycles that have been collected could be extended to more than thirty new cycles that have not been described yet [34]. The metabolic clockwork was constructed at the Semmewes Medical School, Budapest, by means of the Biochemical Pathways, Richard Michal, Editor, Third edition, part 1, which is used to teach medical students biochemistry.

### Formose Reaction

Monosaccharides represent one of the most important building blocks of life. One of the oldest prebiotic synthetic routes is the formose network, but it is not related to cyanide. The basic reaction within the formose reaction is aldol addition, in which the aldehyde group helps the extension of the carbon chain. Cyanide reacts with aldehyde and inhibits aldol addition. Hydrogen cyanide and formaldehyde contribute to the formation of complex nucleobases, which are components of RNA [19]. The formose reaction became synonymous with sugar production reflecting the opinion of those scientists who see the formose reaction as the best candidate for the prebiotic synthesis of sugars [35]. At the prebiotic time (4.5–4 billion years ago), the conditions in the Hadean could have been suitable for the formose reaction despite its messy alkaline reactions and link to metabolism [35].

Butlerow and Omran’s group think that the formose reaction should not be solely focused on the use of sugars for genetic materials, but also on the origins of metabolism via metabolic molecules [21,33]. The formose process consists of two sets of reactions [21]. The first set is slow, taking place at a high concentration of formaldehyde to form glycolaldehyde. The second reaction is fast and autocatalytic, involving the formation of several aldoses and ketoses in the presence of low molecular weight substrates (formaldehyde, hydrogen cyanide). Despite the messy alkaline reactions, Omran’s group sees the RNA World hypothesis and the formose reaction as the best candidates for the prebiotic synthesis of sugars [33]. Bernhardt thought that the RNA World hypothesis was among the worst theories on the early evolution of life [33]. Nevertheless, the idea of Berhardt that the phosphorylation of ribose-to-ribose phosphate and the polymerization of ribose phosphates to the ribose phosphate backbone significantly contributed to the idea of abiotic ribose formation [34].

## 3. The Plausibility of the Formose–Ribose–RNA Pathway

Those reactions that contributed to the formation of prebiotic RNA have been selected. More importantly, this section deals with observations that are closely related to the origin of life and confirm the plausibility of the formose–ribose–RNA pathway.

The plausibility of the formose–ribose–RNA reactions has been tested through prebiotic processes that required a reliable source of free energy. The formose reaction is a potential energy source for sugars. At moderate to elevated temperatures and pH ranges, these sugars such as glucose, ribose and other monosaccharides are produced in the presence of CaCO_3_ [33]. Originally, it was postulated that life on Earth was formed in a warm pond [35]. The energetically and minerally rich as well as diverse hydrothermal vents exhibited a broader environmental spectrum acknowledged in prebiotic chemistry [36,37,38]. During the emergence of life according to the RNA World theory, sugars played an important role in the formation of biopolymers and the control of energy flow [30,39,40]. Moreover, the sugar-producing formose reaction is likely to have occurred not only on Earth [41] but also in interplanetary space and cosmic dust clouds (planets) [42,43,44,45].

Despite intensive efforts, how life came into being cannot be revealed, yet. Nevertheless, unknown metabolic pathways and early metabolic steps in evolution will be reconstructed, as exemplified by this attempt and supported by the early appearance of life on Earth, the conservation of basic principles of life and the biological universality of chemical reactions under our planetary conditions [46]. The plausibility of the formose reaction in alkaline hydrothermal vent environments has been tested [47]. It was confirmed that CaCO_3_-based chemical gardens catalyzed the formose reaction to produce glucose, ribose, and other monosaccharides. The work of Omran’s group allowed them to conclude that the formose reaction is involved in a plausible prebiotic formose pathway in alkaline hydrothermal vent environments, favoring the RNA world hypothesis [33,47]. In addition to hydrothermal vents, more general aquatic environments have been proposed [48].

The properties of prebiotic RNA made the RNA World hypothesis of Gilbert plausible but not universally accepted [34] among the theories of the origin of life. Due to its double function, RNA became known as the first genetic molecule and a non-enzymatic catalyst; consequently, the clarification of the prebiotic synthesis of RNA has remained of primary importance. The formose reaction results in many other sugar molecules in ribose, and the attachment of nucleobases in the ribose–phosphate backbone and preRNA. The rest of this review summarizes those reactions that contribute to the probability of there being other steps in the pathway based on the observations of evolutionary scientists. In an earlier review, (i) the formose reaction, (ii) the reactions of ribose phosphorylation and (iii) the formation of RNA were regarded as related but independent reactions [49]. Earlier, the major reason for the objection against the formose reaction serving as a sugar source for RNA synthesis was the low yield of ribose [27,29,50]. The formose reaction as a ribose source was also rejected because it produced an adverse mixture of compounds and took place in an aqueous media, although sugar synthesis in the gas-phase formose reaction was already known [51]. Moreover, the prebiotic synthesis of simple sugars under gas-phase conditions by the interstellar formose reaction has been published [52]. Much higher yields of ribose production in the formose reaction were measured in the 1990s due to agents such as borates, calcium and reactive phosphate minerals stabilizing ribose production [21,23]. Other product stability issues in the formose system were due to higher temperatures and pH conditions [53].

One requirement of an efficient formose reaction is the high concentration of formaldehyde [54]. It is not clear whether the extraterrestrial delivery of formaldehyde was high enough because the abundance of formaldehyde and glycolaldehyde is very low and the type of meteorite that contains these aldehydes contains just 3% in total [55]. Formaldehyde (HCHO) is especially important with respect to the origin of an RNA or pre-RNA world, since HCHO may be a precursor to ribose and other sugars [56]. Another possible source is impacts from the more common meteorites [57]. An experimental investigation of the formation of formaldehyde by Hadean and Noachian impacts was conducted in [58]. The synthesis of monosaccharides under nonaqueous conditions and physical forces such as meteorite impacts and lithospheric activity were catalytically accelerated in [59].

### 3.1. Significance of Ribose Phosphorylation

As far as the phosphorylation issue is concerned, the greatest extent occurred in ribose at the 5′-hydroxyl position compared to ribose 1′-OH and other aldopentoses in the presence of borate resulting in enhanced stability of ribose. Mineral catalysis and borate are assumed to form a complex with ribose by fixing ribose in the furanose form and improving its stability leading to the high yield of regioselective phosphorylation of ribose at its 5-hydroxyl [60,61]. The phosphorylation in the presence of borate preferentially uses pyrophosphate [61]. The polymerization of ribose-phosphate is an open question, and for RNA synthesis through the poly-ribose–phosphate pathway [62].

### 3.2. Attachment of Nucleobases to the Ribose–Phosphate Backbone

The RNA World hypothesis [50] assumes that life on Earth began with the abiotic formation of a simple RNA molecule but does not support the biochemical origin of RNA. This belief turned the interest in the hypothesis toward inorganic chemical reactions [30]. Orgel was the first to suggest that ribose was generated from glycolaldehyde and formaldehyde corresponding to the formose reaction and that nucleobases were coupled to the 5- or 6-membered rings of ribose bringing about ribofuranosides and ribopyranosides [30]. The compounds providing the missing link in Orgel’s pathway were ribose-phosphates [33]. To complete the formation of abiotic RNA, it was assumed that the polymerization of the ribose–phosphate backbone was followed by the attachment of prebiotic purine and pyrimidine bases bringing about preRNA [51], which, however, was not yet a genetic molecule and came into being without the involvement of nucleotide precursors [51]. Similarly to the regioselective synthesis of ribose phosphorylation, the synthesis of purine and pyrimidine nucleobases could have been made stereoselectively under prebiotic plausible conditions [63].

The significance of the formose–ribose–RNA reactions is their relationship to the origin of life, leading from simple abiotic reactions of formaldehyde to complex sugars including ribose, one of the building components of RNA. In conformity with the idea of Butlerow and not focusing only on sugar production [21], the origin of metabolism could have started with the formose reaction, regarded as the beginning of the metabolic pathways. At around 4.5–4 billion years ago, the conditions in the Hadean when the Earth cooled into a volcanic world could have been suitable for the formose reaction [15] and one of theone, if not the earliest, metabolic reactions.

The “RNA first” model for the origin of life has been developed by research suggesting the “Discontinuous Synthesis Model” for the formation of RNA from precursor molecules that might have been available on early Earth from prebiotic reactions [64]. The formose–ribose–RNA pathway conforms to the discontinuous synthesis model by melting together different reactions into the first plausible metabolic pathway.

### 3.3. From preRNA to Genetic RNA

PreRNA is formed from the components of RNA and not from ribonucleotides. The sequence of nucleotides is responsible for its information content. As there are no copied ribonucleotides, preRNA does not contain genetic information but is synthesized in abiotic conditions, thus it is called preRNA. However, once it was formed, the hydrolysis of preRNA followed the degradation pattern of other RNA molecules and generated nucleoside monophosphates (NMPs). Although activated ribonucleotides can polymerize to nucleotides, how has been explained based on plausible reactions [49]. Briefly, the hydrolysis of non-genetic preRNA-generated nucleoside monophosphates were phosphorylated to nucleoside triphosphates. These NTPs could serve as the building blocks of genetic RNA [51].

## 4. Hierarchical Processes Involved in the Transfer of Cellular Information

**Figure 4:**
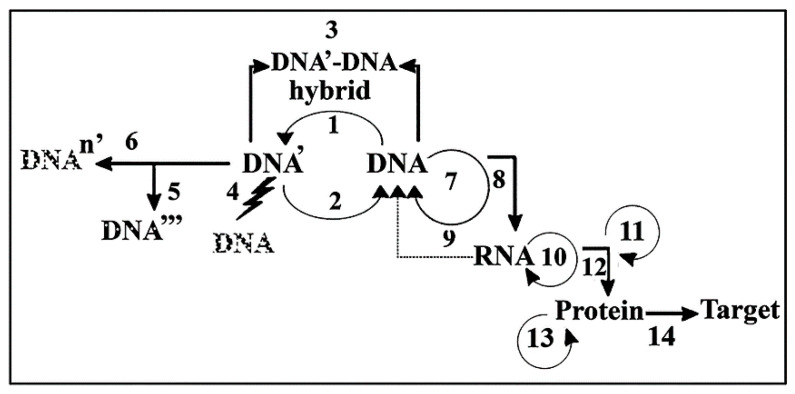
DNA Empire: Hierarchical arrangement of processes involved in the transfer of genetic information from DNA through RNA to proteins. Processes belonging to the intracellular transfer of genetic information are DNA ↔ DNA transfer processes. (1) Mutation: DNA => DNA′. (2) DNA repair: DNA′ => DNA. (3) Recombination: crossover, gene conversion (DNA′-DNA hybridization). Recombination can take place intracellularly but is mainly an intercellular process. (4) Apoptosis (programmed cell death, high levels of DNA damage). (5). Aging, several mutations, persistent DNA damage: DNA′ => DNA″ => DNA‴. (6). Malignant transformation with multiple mutations: DNA => DNAn′ (persistent DNA damage, ma)ny mutations, mutant p53). (7). DNA replication: DNA—DNA reduplication (high fidelity, HiFi process, 1:10^10^ misincorporated deoxyribonucleotide). (8–9). DNA => RNA transfer. (8). Transcription: DNA => RNA (medium fidelity, MeFi process, 1:10^5^ misincorporated ribonucleotide). (9). Reverse transcription: RNA => DNA (in retroviruses). (10). RNA replication: RNA => RNA (in RNA viruses). (11). Processes belonging to posttranscriptional modifications: 5′-cap formation, 3′-polyA formation, splicing. (12). Translation: RNA => protein (low fidelity, LoFi process, 1:10^4^ misincorporated amino acid) (13). Processes belonging to protein modification: protein splicing, transglutamination. (14). Protein targeting: information reaches intra- or extracellular destination [49]. Reproduced with permission from Banfalvi, G. “Prebiotic Pathway from ribose to RNA formation”. *Int. J. Mol. Sci.*, 2021, Figure 5.

Figure 4 represents different levels of processes involved in transferring the genetic information that is known today. At the top, one can find the highest level of transfer of genetic information known as recombination forming DNA′-DNA hybrids followed by processes of DNA-DNA formation at the next lower level. In next lower level are the processes of gene expression and at the lowest level are those processes that are related to the formation proteins. The hierarchical level of processes is known as the DNA Empire where the general rule of genetic information prevails: DNA makes RNA which makes protein. The formose–ribose–RNA metabolic pathway suggests that the formation of ribose preceded preRNA, followed by nucleotides and genRNA synthesis. The prebiotic synthesis of amino acids provided evidence that proteins came second (or nearly the same time as RNA) and DNA arose only as the third genetic molecule showing that ribose and RNA are the molecules of providence, probably not protein or DNA.

## 5. Discussion

The protometabolic reactions that evolved into the metabolic pathways have not been clarified. The chemical origin of biological carbon metabolism may have relied on the versatility of a single molecule, e.g., hydrogen cyanide [65] and other small C1 biogenic compounds (HCO, HCHO, CO_2_) connecting them to long chains resulting in informational macromolecules. During the selection process, it turned out that the units of biological information are the nucleotides that became present on Earth in measurable amounts under prebiotic conditions. Several linear, circular, spiral pathways of arranged biochemical reactions have been deciphered with the notable exception of the synthesis of prebiotic RNA. Among the attractive hypothetical models, the discontinuous synthesis of RNA synthesis is the closest we could get [66]. The discontinuous model is deficient since no experiment has joined together its steps (similarly to other models), creating an ‘asphalt problem’. Moreover, the bonds of the discontinuous model are thermodynamically unstable due to their hydrolysis in water creating the ‘water problem’ [66]. However, in the pathway, the hydrolysis of preRNA to NMPs is an important step for the rephosphorylation to NMPs, generating NTP substrates for genRNA synthesis. It is possible that the discontinuous preRNA synthesis developed in an intermountain dry valley containing evaporating water with a high pH, borate-stabilized carbohydrates and ribose, leading to nucleosides, nucleotides and oligomeric RNA [66]. Our strategy is to collect short metabolic patches of known reactions and fit them into extended, ultimately metabolic, pathways. The formose–ribose–RNA pathway conforms to the criteria of the discontinuous RNA synthesis model.

## 6. Conclusions

Phosphorylated molecules have a wide range of biological functions, suggesting that they may have played important roles in the prebiotic Earth [67]. Phosphate is ubiquitously present on Earth and is regarded as a factor in the origin of life as a central element of life [68]. The formation of organophosphate molecules by prebiotic processes relied on nonenzymatic synthesis [69]. It is likely that ribose served as the abiotic primary source and was phosphorylated to 5′-ribose phosphate for RNA formation, supported by the observation that ribose–phosphate can be polymerized to a ribose–phosphate backbone [33].

The experimental background supports the prebiotic selection of ribose for RNA formation, isolation, nd nucleotide synthesis was based on metal-doped clays [70]. Molecular modelling experiments revealed that ribose was selected as the exclusive sugar component for nucleic acids because it was the only pentose that fitted perfectly into the nucleotide structure [20]. Ribose served as the abiotic primary source and phosphorylated to 5′-ribose phosphate for RNA formation. The formose–ribose–RNA pathway is supported by the observation that ribose–phosphate can be polymerized to a ribose–phosphate backbone [33]. It is assumed that by attaching the nucleobases to the backbone in a random fashion the non-genetic preRNA was formed. To generate a selectable pool of ribonucleoside triphosphates (NTPs), the preRNA was then hydrolyzed to NMPs due to the presence of the reactive cis diols in the structure. NMPs were phosphorylated to NTPs that served as substrates for the genRNA synthesis. NTPs catalyze the polymerization of several processes. The transition of NDPs to dNDPs in the ribonucleotide reductase reaction replaced the 2′C-OH with H, helped to generate dNTPs, and led to DNA synthesis.

The abiotic source for RNA formation in our pathway was ribose produced in the formose reaction even if it assumed that the yield was low but could be improved by mineral phosphates, high temperature and elevated pH [21,23]. Orgel’s skepticism that metabolic pathways and life could not come into existence spontaneously on the early Earth [42] but evolved step by step in a discontinuous manner is shared, as Benner suggested [24]. Ribose–phosphates could be polymerized to the ribose–phosphate backbone [33]. The nucleobases attached to this backbone in a random fashion brought about non-genetic preRNA [51]. To generate a selectable pool of ribonucleoside triphosphates the preRNA was first hydrolysed to NMPs. NMPs were phosphorylated to NTPs that serve as substrates for genRNA synthesis. NTPs can catalyze the polymerization of several processes such as the transition from NDPs to dNTPs to replace 2′C-OH with H and the formation of DNA. The development of the recently known and generally accepted metabolic processes of the transfer of genetic information from the RNA World to the DNA Empire is summarized as the hierarchical arrangement of the processes of the Central dogma of Molecular Biology stating that DNA makes RNA makes protein [71,72]. The general view of the transfer of genetic information conforms with those theoretical and experimental considerations that lead one to believe that a pure world of RNA never existed, rather a mixed world made of covalently linked RNAs with amino acids and peptides [73,74,75,76,77,78,79]. These and other experiments proved that amino acids could be synthesized under abiotic conditions [14,15]. Thus, amino acids and peptides deserve to be mentioned but are not in the focus of this review.

## Data Availability

The author confirms that the data supporting the findings are available within the article.
